# Transgenic microRNA‐14 rice shows high resistance to rice stem borer

**DOI:** 10.1111/pbi.12990

**Published:** 2018-08-24

**Authors:** Kang He, Huamei Xiao, Yang Sun, Simin Ding, Gongming Situ, Fei Li

**Affiliations:** ^1^ Institute of Insect Sciences/Ministry of Agriculture Key Laboratory of Molecular Biology of Crop Pathogens and Insect Pests College of Agriculture and Biotechnology Zhejiang University Hangzhou China; ^2^ College of Life Sciences and Resource Environment Yichun University Yichun China; ^3^ Department of Entomology College of Plant Protection Nanjing Agricultural University Nanjing China; ^4^ Institute of Plant Protection Jiangxi Academy of Agricultural Sciences Nanchang China

**Keywords:** miR‐14, rice stem borer, ecdysone biosynthesis, insect‐resistant, insect pest control

## Abstract

Rice stem borer (RSB,* Chilo suppressalis*) is an insect pest that causes huge economic losses every year. Control efforts rely heavily on chemical insecticides, which leads to serious problems such as insecticide resistance, environment pollution, and food safety issues. Therefore, developing alternative pest control methods is an important task. Here, we identified an insect‐specific microRNA, miR‐14, in RSB, which was predicted to target Spook (*Spo*) and Ecdysone receptor (*EcR*) in the ecdysone signalling network. *In‐vitro* dual luciferase assays using HEK293T cells confirmed the interactions of *Csu‐miR‐14* with *CsSpo* and with *CsEcR*. *Csu‐miR‐14* exhibited high levels of expression at the end of each larval instar stage, and its expression was negatively correlated with the expression of its two target genes. Overexpression of *Csu‐miR‐14* at the third day of the fifth instar stage led to high mortality and developmental defects in RSB individuals. We produced 35 rice transformants to express *miR‐14* and found that three lines had a single copy with highly abundant *miR‐14* mature transcripts. Feeding bioassays using both T_0_ and T_1_ generations of transgenic *miR‐14* rice indicated that at least one line (C#24) showed high resistance to RSB. These results indicated that the approach of miRNAs as targets has potential for improving pest control methods. Moreover, using insect‐specific miRNAs rather than protein‐encoding genes for pest control may prove benign to non‐insect species, and thus is worthy of further exploration.

## Introduction

Rice (*Oryza sativa*) is one of the most widely consumed foods in the world (FAOSTAT, 2018). Unfortunately, rice production suffers severe damage from more than 200 insect pests at different life stages (Cheng, 1996). Among them, rice stem borer (RSB, *Chilo suppressalis* Walker) is one of the most serious insect pests (Bottrell and Schoenly, 2012; Chen *et al*., 2011). Although many methods have been developed for controlling these insect pests, application of chemical insecticides is still the most widely used (Huang *et al*., 2017). Unfortunately, overuse of insecticides has caused several serious problems, such as insecticide resistance, environment pollution, and food safety issues (Chagnon *et al*., 2015). Therefore, developing alternative pest control methods is highly necessary.

Insect‐resistant genetically modified (GM) crops are an increasingly utilized method of pest control (Chen *et al*., 2011). In the mid‐1980s, the first GM cotton expressing insecticidal Crystal (Cry) proteins, derived from the bacterium *Bacillus thuringiensis* (*Bt*), was generated, which provided a new advantage for crops against cotton bollworm (Vaeck *et al*., 1987). In the past two decades, transgenic *Bt* cotton has been widely adopted as a GM insect‐resistant crop (Li *et al*., 2016). In China, dozens of *Bt* rice and *Bt* maize lines have been developed, some of which showed high efficiency in controlling target lepidopteran pests on rice (Liu *et al*., 2016). Botanically derived insecticidal genes, such as *Galanthus nivalis* agglutinin (GNA; Rao *et al*., 1998), and protein inhibitor genes (e.g., *pin II*,* CpTi*,* SKTI* and *BTI‐CMe*) were also used to produce various GM crops resistant against to herbivory planthoppers, stem borers and rice water weevils (Alfonso‐Rubi *et al*., 2003; Duan *et al*., 1996; Lee *et al*., 1999; Xu *et al*., 1996).

A breakthrough in pest control involving RNA interference (RNAi) occurred in 2007. Transgenic crops expressing double‐stranded RNA (dsRNA) against a suitable target gene were shown to exhibit resistance to insect pests (Baum *et al*., 2007; Mao *et al*., 2007). Since then, an increasing number of reports demonstrated that repressing insect genes by expressing a small RNA normally expressed in plants conferred protection against insect pests (Auer and Frederick, 2009; Ossowski *et al*., 2008). The success of transgenic crops using RNAi significantly broadened the scope of target genes that can be used for pest control (Huvenne and Smagghe, 2010; Price and Gatehouse, 2008). Much effort has been devoted to finding more suitable insecticidal target genes. Most of the newly identified targets for RNAi pest control are protein‐encoding genes (Zhang *et al*., 2013). However, non‐coding RNA genes have seldom been used to control insect pests.

MicroRNAs (miRNA) are a class of small, non‐coding RNAs that down‐regulate the targets by degrading messenger RNA (mRNA) transcript or repressing translation, mainly by targeting the 3′‐ends of the transcript (Huntzinger and Izaurralde, 2011). In insects, miRNAs have been reported to regulate metamorphosis in development (Bushati and Cohen, 2007; Ylla *et al*., 2016). As a result of the evidence indicating the important roles of miRNAs in regulating insect development, researchers began to explore the application of miRNA in pest control. The larvae of cotton bollworm, *Helicoverpa armigera*, that fed on bacteria expressing an artificial miRNA (amiRNA) sequence specifically targeting the ecdysone receptor (*EcR*) gene showed greater mortality, developmental defects, and a significant decline in reproductive ability (Yogindran and Rajam, 2016). Feeding on transgenic tobacco plants producing an amiRNA based on endogenous *miR‐24* against a chitinase gene significantly reduced the level of *chitinase* transcripts in *H. armigera* larvae, causing cessation of molt (Agrawal *et al*., 2015). Although RSB larvae that were continuously fed with transgenic *Csu‐novel‐miR‐15* rice showed a 4‐day delay of pupation, no significant lethal effect was observed (Jiang *et al*., 2016), the target genes and biological function of this novel miRNA remain unclear.

Insect‐specific miRNAs present a promising target for pest control that may have less negative impacts on the environment and non‐insect species than chemical pesticides. Here, we identified an insect‐specific miRNA, *Csu‐miR‐14,* that targets two genes, *CsSpo* and *CsEcR*, in the ecdysone signalling pathway of *C. suppressalis*. *In vitro* and *in vivo* experiments confirmed the interactions between *Csu‐miR‐14* and its molecular targets. We produced transgenic rice expressing artificial miRNA (*amiR‐14*). Feeding on transgenic *miR‐14* rice led to a significantly higher mortality and developmental defects in RSB.

## Results

### *CsSpo* and *CsEcR* are putative targets of *Csu‐miR‐14*

**Figure 1:**
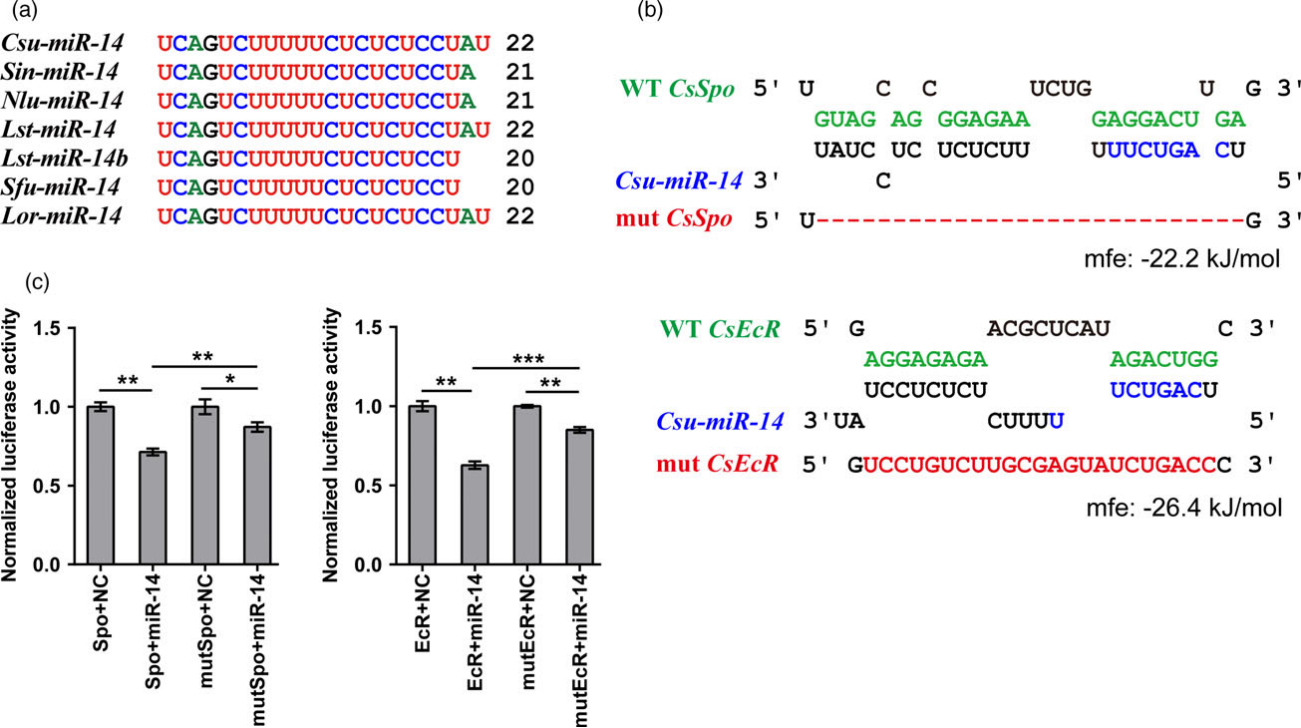
Insect‐specific miRNA *miR‐14* and its two predicted targets in rice stem borer (RSB). Spook (*Spo*) and ecdysone receptor (*EcR*) are two genes in the ecdysone signaling network. (a) Mature sequence alignment of *miR‐14* among rice pests. Csu, *Chilo suppressalis*; Sin, *Scirpophaga incertulas*; Nlu, *Nilaparvata lugens*; Lst, *Laodelphax striatellus*; Sfu, *Sogatella furcifera*; Lor, *Lissorhoptrus oryzophilus*. (b) Sequence alignment of *Csu‐miR‐14* with the predicted target sites in the 3′UTR of *Spo* and *EcR*. Mutants (mut) were generated by deletion (red dash) or mutating (red letters) of complementary bases in the binding region of *Csu‐miR‐14*. Seed sequences were indicated in purple. Wild‐type (WT) sequences of those mutations are indicated in green. Mfe, minimum free energy of the binding between miRNA and UTRs predicted by RNAhybrid. (c) Dual‐luciferase activities compared between *miR‐14* with negative control (NC) in HEK293T cells (*n *=* *6). The data are shown as means ± SE, *P <* *0.05, **P <* *0.01, ***P <* *0.001

We downloaded 2,474 insect miRNA sequences from miRbase 22.0 and 517 miRNAs from publicly reported references (Chang *et al*., 2016; Yang *et al*., 2017), covering 14 species across five insect orders. To amass more insect miRNA information, we sequenced small RNA libraries of three notorious insect pests, *Scirpophaga incertulas* (yellow stem borer, YSB), *Nilaparvata lugens* (brown planthopper, BPH) and *Laodelphax striatellus* (small brown planthopper, SBPH), yielding 76, 110 and 118 miRNAs, respectively (Tables S1 and S2). Conservation analysis of all miRNAs from 17 insect species identified an insect‐specific miRNA, *miR‐14*, which was conserved among stem borers, and planthoppers (Figure 1a). Then, we focused on RSB to study the function of *miR‐14* and its potential application in pest control. To predict the targets of *Csu‐miR‐14*, we identified the 3′ UTRs of all genes with a customized Perl script from the transcriptomes and genome of RSB. Then, we predicted the targets of *Csu‐miR‐14* by using miRanda (John *et al*., 2004) and RNAHybrid (Kruger and Rehmsmeier, 2006), which indicated that two genes in the ecdysone signalling network, *CsSpo* and *CsEcR*, were putative targets of *Csu‐miR‐14* (Figure 1b). Target prediction in other insect pests suggested that *miR‐14* also targeted genes in the ecdysone signalling network (Table S3), indicating that *miR‐14* regulation of the ecdysone signalling network is a conserved process among insects. We used an *in vitro* dual‐luciferase reporter assay to validate the interactions between *Csu‐miR‐14* and its target genes. The 3′‐UTRs containing the predicted target sites of *CsSpo* and *CsEcR* were cloned into the downstream of luciferase genes in the pMIR‐REPORT vector (Obio, China) and then transfected into HEK293T cells. The results indicated that luciferase activities were significantly reduced in the presence of *Csu‐miR‐14* mimics (*P *<* *0.05, Student's *t*‐test; Figure 1c), confirming the interactions of *Csu‐miR‐14* with each of its two targets, *CsSpo* and *CsEcR*.

### The expression of *Csu‐miR‐14* negatively correlated with the expression of *CsSpo* and *CsEcR*

**Figure 2:**
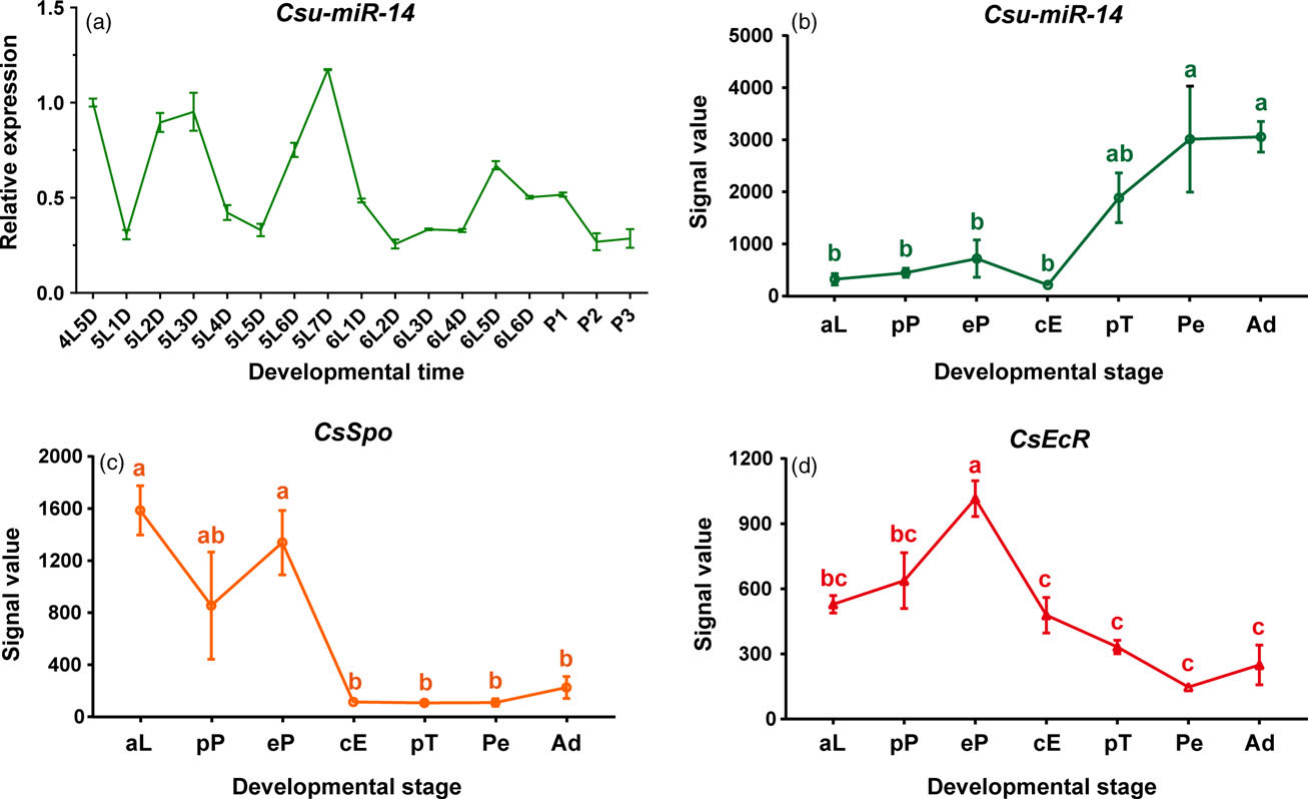
Expression profiles of *Csu‐miR‐14* and its two target genes, *CsSpo* and *CsEcR* at different developmental stages. (a) qRT‐PCR analysis of *Csu‐miR‐14* from the fifth day of the fourth‐instar larvae (4L5D) to the third‐day pupa (P3) stage, indicating that *Csu‐miR‐14* is highly expressed at the end of each larval instar stage. (b) The changes in expression levels of *Csu‐miR‐14*, (c) *CsSpo*, and (d) *CsEcR* estimated by a previous microarray analysis at seven developmental stages of RSB (He *et al*., 2017; Sun *et al*., 2017) including aging larvae (aL), prepupae (pP), early pupae (eP), compound eye formation stage (cE), pupae elongation stage (Pe), and adult (Ad). The data are shown as means ± SE (*n *=* *3). Signal values were compared by one‐way ANOVA among various stages and significant difference was indicated with different letters after Turkey's multiple comparison test.

Expression analysis indicated that the level of transcripts of *Csu‐miR‐14* were high at the end of each instar and then declined sharply in the initial point of the following stage (Figure 2a). In our previous report (He *et al*., 2017; Sun *et al*., 2017), customized microarrays were designed to study the expression of small RNA and mRNA from the same batch of RSB samples including the stages of ageing larval, prepupal, early pupal, compound eye formation, pretarsal formation, pupal elongation and adult. This enabled us to calculate the co‐expression patterns between *Csu‐miR‐14* and its target genes. Poisson correlation analysis showed that the abundance of *Csu‐miR‐14* was negatively correlated with the abundances of *CsSpo* (*r *= −0.614, *P  *=* *0.143) and *CsEcR* (*r *=* *−0.714, *P *=* *0.072; Figure 2b–d), implying that the role of *Csu‐miR‐14* is to eliminate the functions of ecdysone.

### *Csu‐miR‐14* controls metamorphosis development of RSB

**Figure 3:**
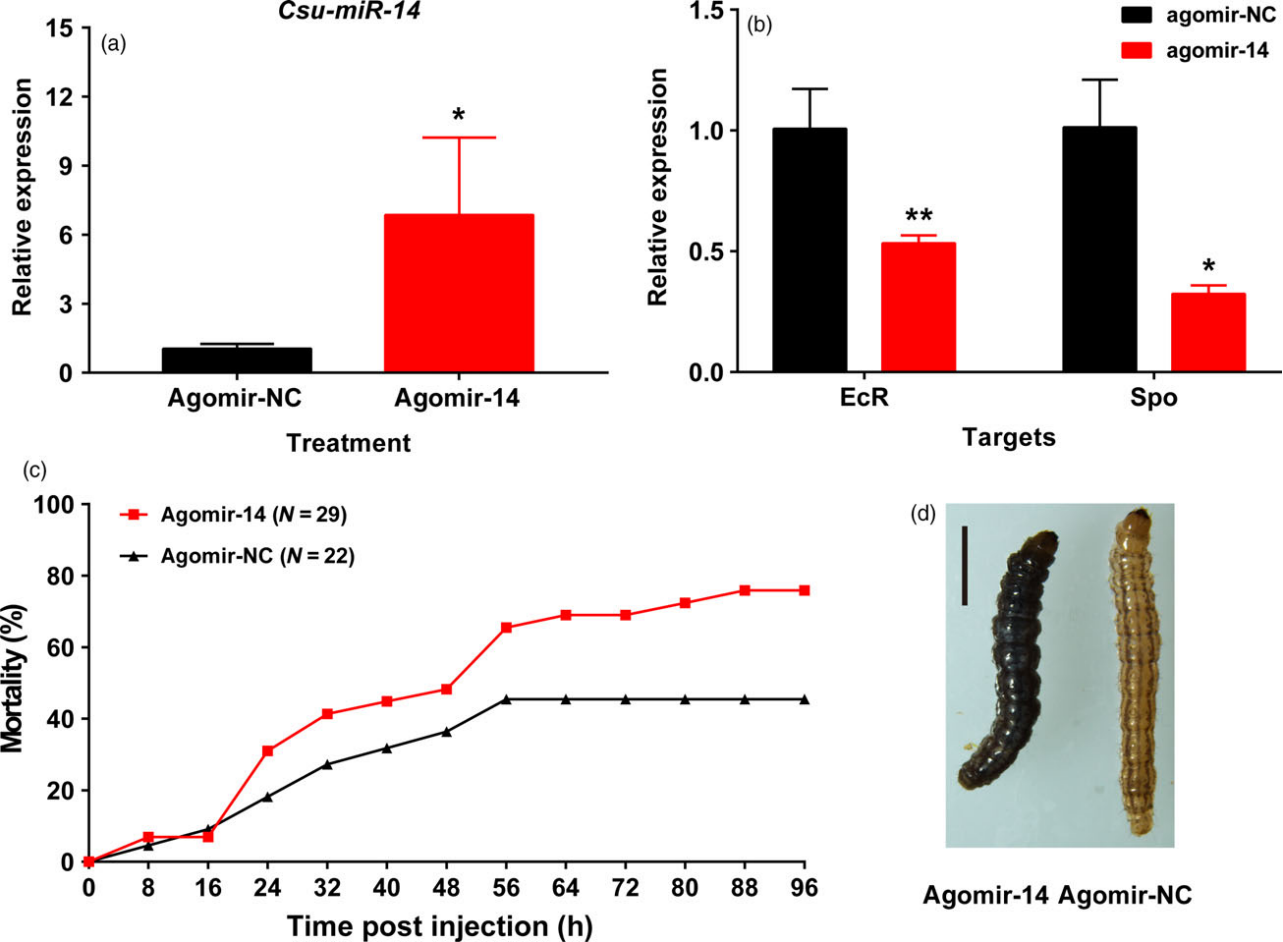
Overexpression of *Csu‐miR‐14* led to high mortality and developmental defects in RSB. (a) The transcript abundance of *Csu‐miR‐14* significantly increased at 24 h after injection of agomir‐14 (Student *t*‐test, *P <* *0.05, **P <* *0.01). (b) The transcript abundance of target genes *CsSpo* and *CsEcR* were significantly reduced at 24 h post‐injection. (c) Overexpression of *Csu‐miR‐14* led to higher mortality from 24 h to 96 h post‐injection. (d) The abnormal phenotype of the agomir‐14 treated group compared to the control.

To study the *in vivo* function of *Csu‐miR‐14*, we overexpressed it by injecting the miRNA mimic (agomir‐14) into third‐day larvae at fifth‐instar stage (5L3D) of RSB. Chemically synthesized agomir‐14 (100 pmol) was injected into each 5L3D individual. The abundance of *Csu‐miR‐14* was significantly greater, by 6.86 ± 3.36‐fold at 24 h post‐injection than in the control (Figure 3a, Mann–Whitney *U*‐test, *P *<* *0.05), whereas the expression of the two targets, *CsEcR* and *CsSpo*, was lower than their corresponding controls by 46.6% (Student's *t*‐test, *P *<* *0.05) and 67.7% (Student's *t*‐test, *P *<* *0.05), respectively (Figure 3b). Moreover, a significantly higher mortality was observed in the agomir‐14‐treated group than in the control group from 24 to 96 h post‐injection (Figure 3c). The individuals treated with agomir‐14 showed an abnormal phenotype with developmental defects such as an unusually dark body colour (Figure 3d).

### GM rice expressing mature *Csu‐miR‐14*

**Figure 4:**
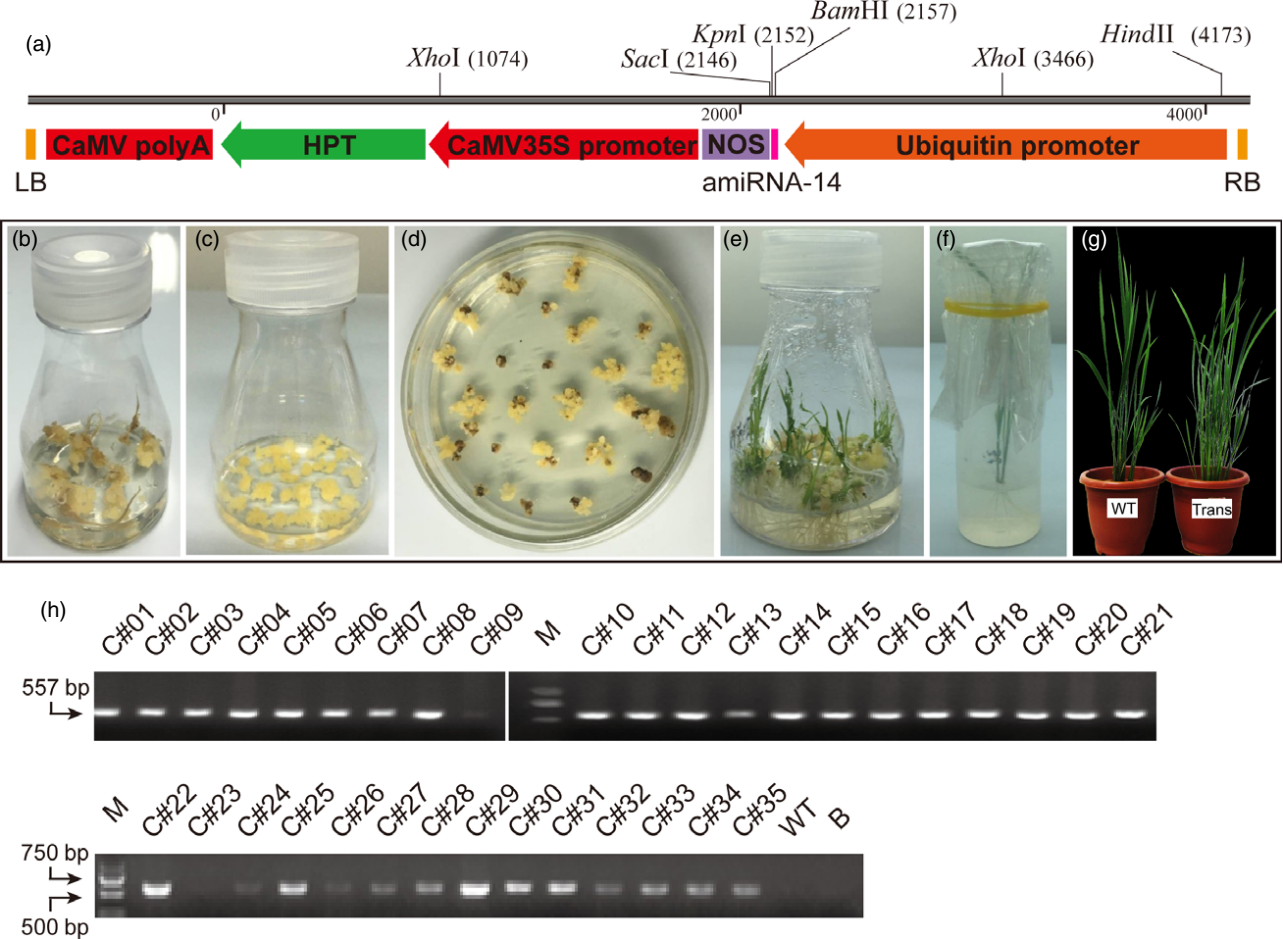
Genetically modified rice expressing *miR‐14* and PCR validation of rice transformants. (a) Schematic diagram of *amiR‐14* expression cassette pUbi‐amiR‐14 introduced into rice variety ZH11 via *Agrobacterium*‐mediated transformation. Pink box indicates amiRNA‐14 precursor. *Hpt*, hygromycin B phosphotransferase; NOS, Nos terminator; RB, right border; LB, left border. (b) Embryonic callus induction of the seeds processed by shelling and disinfection. (c) Subculture of the callus grown in the dark for 20 days. (d) Screening for positive rice transformants by kanamycin. (e) Seedlings from resistant callus at 40 days. (f) Callus rooting for 20 days in the climate incubator. (g) Seedlings were transferred into a greenhouse and grown to obtain the T_0_ generation. WT, wild type plants; Trans, transgenic plants. (h) Selection of the positive rice transformants based on PCR amplification of the *hpt* gene. M, DL2000 marker; B, blank control.

The mature sequence of *Csu‐miR‐14* was used to design the miRNA expression cassette *ubi*::*amiR‐14*‐*nos‐hpt* (Figure 4a), with the expression of artificial *miR‐14* driven by a ubiquitin promoter. The *amiRNA* vector was introduced into embryonic callus of *japonica* rice variety Zhonghua (ZH11) via Agrobacterium‐mediated transformation (Figure 4b), yielding 35 rice transformants after kanamycin screening. The seedlings then were transplanted into pots of soil and grown in the greenhouse (Figure 4c–g). To confirm the insertion of the *amiR‐14* expression cassette in the genome of GM rice, a pair of primers was designed to amplify a 557‐bp fragment of the selective marker gene hygromycin B phosphotransferase (*Hpt*). Positive PCR products were amplified from 33 transformants, resulting in a transformation efficiency of 94.29% (Figure 4h).

**Figure 5:**
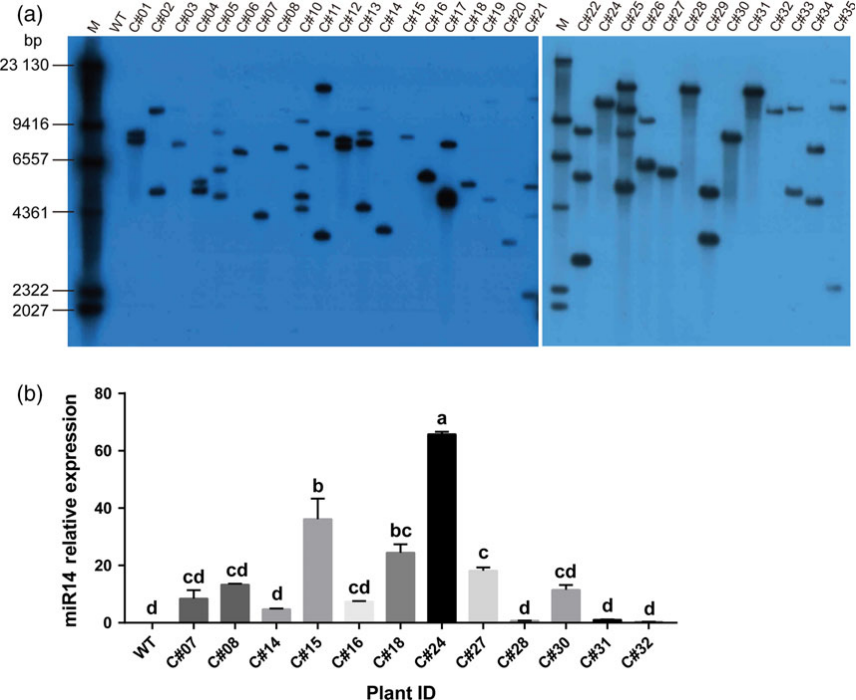
The copy number and mRNA expression for *miR‐14* detected in the T_0_ generation of rice. (a) Southern‐blot detection with a 557‐bp probe against the *hpt* gene. M, λ DNA marker. (b) Relative mRNA abundance of miR‐14 detected with qRT‐PCR. Significant differences (*P *<* *0.05) are indicated with different letters detected by one‐way ANOVA analysis after Turkey's multiple comparison test.

A southern blot was used to determine the copy number of the *amiR‐14* transgene expression cassette in the GM rice genome. A 557‐bp DNA probe was designed based on the complementary sequence of *Hpt*. The results showed that 12 of the 33 positive transformants were detected with a single copy of *amiR‐14*, 11 were detected with two copies, four were detected with three copies, five were detected with four copies, and one was detected with eight copies (Figure 5a). The expression of *amiR‐14* was measured by qRT‐PCR in T_0_ GM rice (Table S4). The highest levels of expression were observed in three transformants, C#22 (205.17 ± 9.40), C#25 (149.02 ± 12.46) and C#24 (65.79 ± 1.47). However, Spearman's correlation analysis showed that there was no positive relation between the expression of *amiR‐14* and its copy number in the GM rice genome (*r *=* *0.172). Observing that transgenic rice with a single copy and high expression of *amiR‐14* can potentially be effective in pest control applications (Figure 5b), we chose three GM rice lines, C#24, C#15 and C#18, for further analysis.

### Feeding on transgenic *amiR‐14* rice led to high mortality and developmental defects in RSB

**Figure 6:**
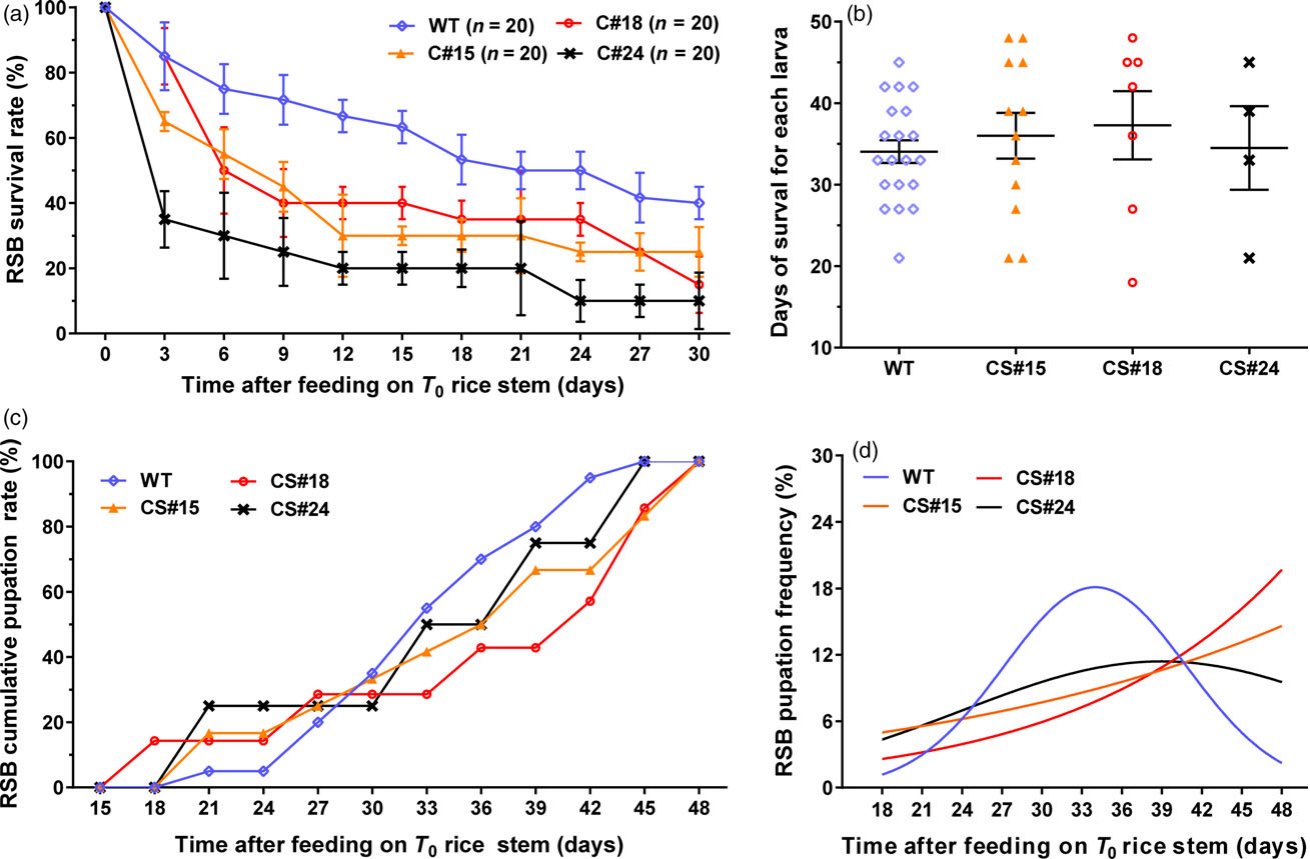
The bioassay of RSB response to ingestion of three different lines of T_0_ transgenic rice plants or the wild‐type. (a) Survival rates, (b) The days of survival for each larva. (c) Cumulative pupation rates. (d) The pupation frequency, where the naturally occurring peak indicating synchrony of pupation among individuals is notably lacking in the transgenic rice groups.

To measure insect resistance in transgenic *amiR‐14* rice, the stems of three positive transformants, C#24, C#15 and C#18, were used to feed first‐instar larvae of RSB. The *japonica* rice variety ZH11 was used as the negative control. The results indicated that the survival rates of groups fed with transgenic *amiR‐14* were significantly lower than that of the control group. The average mortality of the control group was 20% at 7‐day after treatment. However, more than 60% of the tested larvae were dead at 3 days after feeding on C#24, while mortality of the C#24 group reached >80% at 12 days after treatment with GM rice (Figure 6a), showing a relatively high resistance to RSB. The mortalities of the groups fed with C#15 and C#18 were about 60% at 9 days after feeding on GM‐rice, suggesting that they have moderate resistance to RSB (Figure 6a).

In addition, the survivors in the groups fed on GM rice exhibited apparent developmental defects. The larvae feeding on GM rice of the C#15 and C#18 lines had a slightly longer lifespan (36.0 ± 2.8 days for C#15; 37.2 ± 4.2 days for C#18) than the other two groups, but without significant difference. The average lifespan of the C#24 group was 34.5 ± 5.1 days, which was similar to that of the control (34.1 ± 1.4 days; Figure 6b). Compared to the control group, 30% of the survivors that fed on GM rice showed early pupation, while the remaining 70% was delayed in pupation (Figure 6c). The control population showed a peak in the frequency of pupating individuals within a similar time period, which corresponds to the natural feature of developmental synchrony that ensures concurrent sexual maturation of adult females and males. However, this peak did not appear in GM‐rice treated groups (Figure 6d), suggesting that the deaths of the majority of RSB individuals caused by ingestion of the transgenic rice would likely hamper population growth.

### Transgenic *amiR‐14* rice showed high resistance to RSB

**Figure 7:**
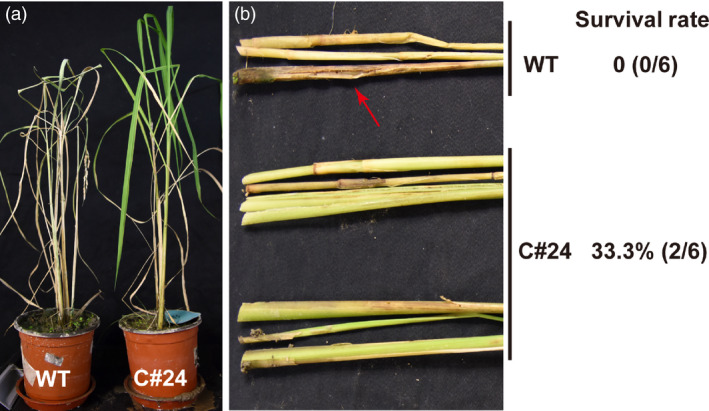
The bioassay of insect resistance in C#24 T_1_ transgenic rice plants exposed to 30 RSB individuals. (a) GM rice C#24 showed high resistance to RSB. (b) There was no obvious tissue damage observed in C#24 GM stems caused by RSB. Red arrow indicates damage of worm holes.

Given the observation that line C#24 had the highest resistance to RSB in feeding assays, we focused on this GM rice line for further analysis. The transgenic line C#24 was grown in the greenhouse and was harvested for seeds of the T_1_ generation. The germination rate of the seeds of line C#24 was 87.5%. Six T_1_‐generation plants of C#24 were selected for estimating their resistance to RSB. The *japonica* rice variety ZH11 was used as the negative control. Thirty first‐instar larvae were transferred to each plant. 50 days later, all ZH11 rice plants were dead with 100% dead hearts because of the extensive damage caused by RSB, whereas all six transgenic *miR‐14* rice plants in two replicates were alive and lacked any indication of damage by RSB herbivory (Figure 7).

## Discussion

Application of transgenic insect‐resistant crops has been shown to be an efficient method for pest control (Vaeck *et al*., 1987). The widely used transgenic *Bt* cotton is a successful example of controlling insecticide‐resistant insect pests (Wu *et al*., 2008). Many insect‐resistant *Bt* rice lines have been developed, although they are not commercially planted so far (Liu *et al*., 2016). Transgenic *Bt* rice showed high resistance to early‐instar larvae of RSB (90%–100% mortality; Liu *et al*., 2016), however, the mortalities significantly decreased when feeding it to higher instar larvae (Hu *et al*., 2005). At present, only a limited number of insecticidal genes have been identified at present (Tabashnik and Carriere, 2017). Here, we demonstrated that an insect‐specific miRNA, *miR‐14*, can be used for producing transgenic insect‐resistant rice. At least one line of transgenic *miR‐14* rice, C#24, showed high resistance to RSB. *miR‐14* has been reported to regulate metamorphosis in a variety of insects (Jayachandran *et al*., 2013; Liu *et al*., 2013; Varghese and Cohen, 2007). Overexpression of *Csu‐miR‐14* led to the death of RSB individuals on such plants which indicates that *Csu‐miR‐14* can interfere with normal metamorphosis development in RSB. The role of *Csu‐miR‐14* is to eliminate the functions of ecdysone after molt. Its first effect is to clean unwanted transcripts of *CsSpo* to prevent the biogenesis of ecdysone; the second effect is to repress *CsEcR*, the ecdysone receptor. Using miRNA in developing transgenic insect‐resistant rice lines significantly broadens the scope of target genes that can potentially be used for pest control.

RNAi is a promising genetic method for pest control (Huvenne and Smagghe, 2010; Price and Gatehouse, 2008). Suppressing the expression of genes participating in important physiological processes may prove lethal to insect pests (Baum *et al*., 2007; Mao *et al*., 2007). RNAi has an advantage over previous transgenic methods in that it can significantly enlarge the scope of target genes that can potentially be used for pest control. At present, most target genes used in RNAi pest control are protein‐encoding genes (Zhang *et al*., 2013). Here, we showed that noncoding RNA genes also can be used for RNAi pest control. Moreover, the ability to select from a wider variety of target genes implies that we are more likely to develop safer alternatives to chemical pesticides by using insect‐specific miRNAs that cannot affect non‐target species because its homologs may not be found in non‐insect species.

Insect metamorphosis is a successful life‐history strategy for fully exploring various environmental conditions (Truman and Riddiford, 1999). The steroid hormone 20‐hydroxyecdysone (20E) coordinates with juvenile hormone (JH) to regulate moulting (Dubrovsky, 2005; Jindra *et al*., 2013; Liu *et al*., 2018; Yamanaka *et al*., 2013). Interfering with ecdysone biogenesis can result in morphological defects (Neubueser *et al*., 2005), and thus can be used for developing pest control methods (Luan *et al*., 2013). The genes in the ecdysone pathway and the miRNAs targeting these genes are promising targets of RNAi pest control, as evidenced by results of this study.

**Figure 8:**
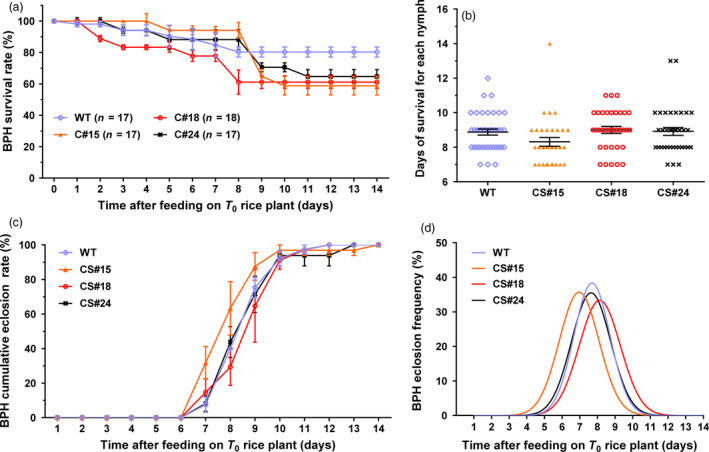
The bioassay of BPH in response to being fed on T_0_ transgenic or wild type rice. Transgenic *miR‐14* rice did not exhibit strong resistance to BPH herbivory. (a) Survival rates of BPH that fed on transgenic rice stems. (b) The days of survival for each nymph. (c) Cumulative eclosion rates. (d) Eclosion frequency.

We also investigated the control effect of transgenic *miR‐14* on BPH. Feeding on transgenic *miR‐14* rice only caused a moderate degree of mortality in BPH, not significantly different from the control (Figure 8), suggesting that the transgenic *miR‐14* rice did not have resistance to BPH, which has piercing‐sucking mouthparts. We suggested that this might be due to only a limited number of *miR‐14* transcripts expressed in the phloem sap of the rice plant. Actually, improving the efficiency of controlling pests with piercing‐sucking mouthparts is still an important task. A recent study showed that less caterpillar damage in *Bt* rice attracts fewer planthoppers, implying possible ecological resistance against BPH (Wang *et al*., 2018). Whether transgenic *miR‐14* rice also has this kind of resistance to nontarget planthoppers requires further investigation.

## Experimental procedures

### Insect rearing

Rice stem borer larvae were maintained on newly germinated rice seeds in glass bottles at 28 ± 1°C with a relative humidity of 70%–80% as described in He *et al*. (2017). Nymphs of *N. lugens* and *L. striatellus* also were maintained on rice seedlings. All of these rice pests were cultured under the same environmental conditions, including a photoperiod of 16 h light/8 h dark.

### Small RNAs sequencing and identification

Total RNA was isolated from samples consisting of a mix of the eggs, larvae (nymphs), pupae and adults for each species (*S. incertulas*,* N. lugens* and *L. striatellus*) using TRIzol (Invitrogen, Carlsbad, CA) reagent following the instructions of the manufacturer. Small RNA sequencing was performed as described in He *et al*. (2017). Purified RNA fragments were first separated by polyacrylamide gel electrophoresis (PAGE) and then ligated to two adaptors (5′/3′) for PCR primers binding. Then these small RNAs were amplified with RT‐PCR, followed by sequencing with an Illumina HiSeq 2000 to construct RNA libraries. After quality control, the cleaned data were used for further analysis. Data statistics from library sequencing are provided in Table S1.

To identify small RNAs in these three pests, cleaned reads were annotated by Blastn against the nr database (https://www.ncbi.nlm.nih.gov), Rfam (Kalvari *et al*., 2018) and RepBase (Jurka *et al*., 2005), and then two algorithms were used to identify the miRNAs. One algorithm involved homology‐searching against arthropod miRNAs in the miRbase (Kozomara and Griffiths‐Jones, 2014) using a cut‐off of 0‐2 nt mismatches or deletions. The other algorithm identified both conserved and novel miRNAs with miRDeep (An *et al*., 2013) against the respective genome sequence of *N. lugens* (Xue *et al*., 2014) and *L. striatellus* (Zhu *et al*., 2017) with the default parameter of ignoring reads of length <18 nt and mapping to no more than 5 genomic loci. For *S. incertulas*, the *C. suppressalis* genomic sequence (Yin *et al*., 2014) was used as the reference. MicroRNA sequences identified by the two methods were pooled, and the redundancies were removed to generate a final set of miRNAs for the three rice pests.

### Target prediction of miR‐14

To predict targets of *miR‐14* in rice pests, the 3′ UTR sequences of 19 genes in the ecdysone signalling pathway network were obtained by searching the genome and transcriptome of all four rice pests using a customized Perl script. Two miRNA target prediction methods, miRanda (John *et al*., 2004) and RNAHybrid (Kruger and Rehmsmeier, 2006), were used to predict the targets of *miR‐14* with the default parameters. The cut‐off for free energy was <−18 kJ/mol. Targets predicted by either algorithm were used for further analysis.

### Cell culture and luciferase assay

The HEK293T cell line has been commonly used in the luciferase assay for the largely conserved mechanisms underlying the processing of pre‐miRNAs between mammalian and insect cells (Bogerd *et al*., 2014; Liu *et al*., 2017). Cells were maintained at 37 °C with 5% CO_2_ in DMEM high‐glucose medium (Gibco, Grand Island, NY) containing 10% foetal bovine serum (Gibco). 3′UTR fragments of the *CsSpo* and *CsEcR* genes were cloned into the pMIR‐REPORT vector (Obio, China) between the firefly luciferase open reading frames (ORF) and the SV40 polyA signal. Cells were transfected with the mixture of 200 ng of the reporter plasmid, and 10 ng of the pRL‐CMV control plasmid in 0.25 mL Lipofectamine^®^ 2000 Transfection Reagent (Invitrogen) in 5‐mL Opti‐MEM^®^ I Reduced Serum Medium (Gibco) in each well of a 96‐well plate. The miRNA mimics were synthesized by RiboBio (Guangzhou, China) and diluted to a concentration of 100 nm. The Dual‐Luciferase^®^ Reporter (DLR™) Assay System (Promega, Madison City, WI) was performed 24 h after transfection according to the manufacturer's protocol. The experiment was performed in six independent replicates. The mean of the relative luciferase expression ratio (renilla luciferase/firefly luciferase) of the control was set to 1.

### MicroRNA agomir and antagomir treatment *in vivo*

Mimics of miR‐14 (agomir‐14) and a negative control (agomir‐NC) were synthesized by RiboBio. For each individual insect, 100 nmol of miRNA mimic (agomir‐14) was injected at the intersegmental membrane in the abdomen of larvae. A total of 30 insects was injected for each treatment. Then, 24 and 48 h after the injection, larvae were flash‐frozen in liquid nitrogen and stored at −80 °C before measurement of mRNA levels.

### Vector construction, rice transformation and screening for expression of amiRNA

Genetic transformation of rice was entrusted to Towin Biotechnology (Wuhan, China). Expression vectors for amiRNA‐14 were designed and constructed according to the sequence of *Csu‐miR‐14*. The amiRNA precursors were generated based on the precursor of rice miRNA *Osa‐miR‐528* (Warthmann *et al*., 2008). Subsequently, the 554‐bp amiRNA precursors were cloned using the pEASY‐T3 Cloning Kit (TransGen, Beijing, China). After validation by sequencing, the amiRNA precursors were released from the T‐vector by digestion with *Bam*HI and *Kpn*I restriction enzymes and cloned into the plant expression vector pC1300‐Ubi‐nos (Chen *et al*., 2013), between the maize ubiquitin promoter and the Nos terminator, to form the final amiRNA expression vectors. The expression vectors were introduced into the *japonica* rice variety ZH11 via *Agrobacterium*‐mediated transformation following the protocol of Lin *et al*. (2002). After transformation and 3 days in culture, calluses were washed three times with distilled water and dried on absorbent paper. Then, calluses were pre‐selected with kanamycin and transferred for differentiation and rooting. Regenerated plantlets were cultivated in a greenhouse for selection.

### Selection of amiR‐14 T_0_ GM rice plants

Genomic DNA was isolated from the leaves of T_0_ GM and wild‐type ZH11 (WT) rice plants according to the CTAB‐based protocol (Doyle and Doyle, 1987; Porebski *et al*., 1997). To identify positive T_0_ transformants, the hygromycin B phosphotransferase (*hpt*) gene was detected with PCR using the genomic DNA as a template. The PCR reaction included a mixture of dNTPs, 0.2 U Takara *rTaq* DNA polymerase, and the primers which are listed in Table S5 (Hpt557‐F, Hpt557‐R). The PCR conditions were as follow: initial denaturation at 94 °C for 3 min; 30 cycles of denaturation at 94 °C for 30 s, annealing at 58 °C for 30 s, and extension at 72 °C for 30 s; and a final extension at 72 °C for 10 min. Genomic DNA from WT plants and double‐distilled water were both used as negative controls. The PCR products were analysed by agarose gel electrophoresis.

### Detection of amiR‐14 copies by southern blot hybridization

To determine the copy number of *amiR‐14*, 10 μg genomic DNA isolated from GM and WT rice leaves of 40‐day old seedlings was digested with 15 U of the restriction enzyme *Hin*dIII (NEB) for 16 h at 37 °C, and the digested DNA was resolved on a 0.8% agarose gel for 16 h at 30 V. Then, the electrophoresis gel was blotted onto a Hybond N+ nylon membrane (GE healthcare, Amersham, UK) by overnight capillary transfer. The blot was hybridized with the complementary strand of the *hpt* gene‐based DNA probe at 48 °C. The blot was subjected to photography using X‐ray film by exposure at −80 °C for 4 h in a darkroom.

### qRT‐PCR for mRNA and miRNA

Fifth day of fourth‐instar larvae (4L5D) to third‐day of the pupa (P3) stage RSB were collected for the measurement of expression of *Csu‐miR‐14*. Total RNA was isolated from the whole‐larval insect body of 40‐day old or rice leaves using TRIzol Reagent (Invitrogen) following the instructions. A first‐strand cDNA synthesis kit (Vazyme, Nanjing, China) was used to prepare the oligo (dT)‐primed cDNA. A stem‐loop cDNA synthesis kit (RiboBio) was used to prepare stem‐loop cDNA. The mRNAs and miRNAs were subjected to qRT‐PCR for the respective gene expression assays using AceQ SYBR Green Master Mix (Vazyme) and Bulge‐Loop™ miRNA qRT‐PCR Starter Kit (RiboBio) according to manufacturers’ directions. Expression of the RSB *Actin*,* 18s* rRNA, and rice *U6* snRNA genes were used as the internal controls for data analysis. qPCR was performed on ABI7500 instrument (Applied Biosystems, Foster City, CA, USA). The program for mRNA qPCR was as follows: 95 °C for 30 s at the initial denaturation step; and 40 cycles at 95 °C for 5 s, and 60 °C for 34 s. The program for miRNA qPCRs was 95 °C for 10 min at the initial denaturation step; and 40 cycles at 95 °C for 15 s, 55 °C for 30 s and 70 °C for 34 s. Data were analysed using the 2^−ΔΔCt^ method of relative quantification (Livak and Schmittgen, 2001). Differences of *amiR‐14* expression between treatments were compared by one‐way ANOVA, followed by Turkey's multiple comparison test. All primers used in the present study are listed in Table S5.

### RSB and BPH feeding assay

#### RSB feeding test

After determination of the *amiRNA‐14* expression levels in rice plants using stem‐loop qRT‐PCR, fresh stems of the rice lines with high levels of expression of *amiRNA* were harvested at the booting stage and cut into 8‐cm segments. Six fresh stem cuttings from each *amiRNA* rice line with 20 newly hatched first‐instar RSB larvae were placed in a Petri dish (9‐cm in diameter). The dish was covered with a piece of moist filter paper and sealed with breathable tape, then incubated in darkness at 28 °C with 80% relative humidity. The rice stems were dissected to find the RSB individuals and fresh rice stems were used to replace old ones every 3 days until all the RSB larvae had developed to pupae, and the mortality of RSB larvae was recorded. Three replications for each *amiRNA* rice line were examined. The data were analysed using Student's *t*‐test to compare RSB mortalities between the groups feeding on ZH11 or transgenic rice.

#### BPH feeding test

Rice stems were individually placed in a glass tube (20‐cm in length and 3‐cm in diameter) for the consecutive BPH feeding test. A fresh rice stem from each amiRNA rice line was infested with 20 newly molted, third‐instar BPH nymphs. The tubes were sealed with nylon mesh to prevent the larvae from escaping and then incubated under the same conditions as described above. The BPH nymphs were inspected twice each day until they emerged as adults. The time‐periods for achievement of developmental stages of the nymphs were recorded.

### Laboratory resistance assay of T_1_ transgenic *miR‐14* rice to RSB

For the field trial assay, the percentage of rice plants with dead‐heart was taken as the main indicator of damage caused by RSB (Liu *et al*., 2016; Shu *et al*., 2000). To examine the resistance of *amiR‐14* transgenic rice plants to RSB in laboratory, 30 newly hatched RSB larvae were transferred to limited numbers of whole rice plants at the booting stage (60‐days old) using a small brush and kept in nylon mesh cages (80‐cm in length and 18‐cm in diameter). Each cage was sealed with glue to prevent the larvae from escaping. The rice plants were grown under the same conditions as mentioned above. Six replications for each *amiRNA* rice line were examined. After infestation, rice plants were checked for white heads or dead hearts caused by RSB every 2 days, and the sheaths were dissected to verify any damage made by RSB.

### Statistical analysis

All data sets are shown as means ± SE (*n *=* *3). The dual‐luciferase reporter assay and quantitative real‐time PCR results were analysed by a two‐tailed unpaired Student's *t*‐test. Significance was set at *P *<* *0.05.

## Author contributions

F.L. designed the work; H.X. and S.D. performed the bioinformatic analysis of miRNA identification, conservation analysis and target predictions. K.H. and S.D. carried out the experiments on miRNA target validation, gene expression, overexpression and feeding assay. Y.S. and G.ST. conducted the experiments for gene expression. K.H. completed and improved the figures. F.L. and K.H. wrote the manuscript.

## Disclosure declaration

The authors declare no conflict of interests.

## Supporting information


**Table S1** Raw reads of three rice pests small RNA libraries.
**Table S2** Gene numbers of miRNAs predicted in three rice pests.
**Table S3** Potential targets of miR‐14 predicted in five rice pests.
**Table S4** Copy number and mRNA expression level of miR‐14 detected in T0 generation transgenic rice plants.
**Table S5** Primers used in the study.
