# Supplementary material for: Transgenic microRNA‐14 rice shows high resistance to rice stem borer
Source: Plant Biotechnol J. 2018 Aug 24;17(2):461–71. doi: 10.1111/pbi.12990 (PMC6335064; doi:10.1111/pbi.12990)
Supplement: Supplementary file 1 — Table S1 Raw reads of three rice pests small RNA libraries. Table S2 Gene numbers of miRNAs predicted in three rice pests. Table S3 Potential targets of miR‐14 predicted in five rice pests. Table S4 Copy number and mRNA expression level of miR‐14 detected in T0 generation transgenic rice plants. Table S5 Primers used in the study. [file PBI-17-461-s001.docx]

Table S1 Raw reads of three rice pests small RNA libraries

| Species | Raw reads | Adapter | Poly A | Insert null | length<18nt | Low quality | Clean reads |
| --- | --- | --- | --- | --- | --- | --- | --- |
| *S. incertulas* | 11,074,074 | 117,225 | 141 | 2,720 | 29,252 | 228,571 | 10,696,165 |
| *N. lugens* | 12,933,341 | 138,969 | 165 | 20,988 | 324,499 | 455,992 | 11,992,728 |
| *L. striatellus* | 12,854,654 | 137,126 | 219 | 10,273 | 884,679 | 54,307 | 11,279,290 |

Table S2 Gene numbers of miRNAs predicted in three rice pests

|  | *S. incertulas* | *N. lugens* | *L. striatellus* |
| --- | --- | --- | --- |
| miRNA by miRBase | 74 | 84 | 87 |
| miRNA by miRDeep | 52 | 53 | 81 |
| Intersection set | 50 | 27 | 50 |
| Novel miRNAs | 2 | 26 | 31 |
| Final miRNAs | 76 | 110 | 118 |

Table S3 Potential targets of *miR-14* predicted in five rice pests

| Species | Genes in ecdysteroid signal pathway | | | | | | | | | | | | | | | | | | | |
| --- | --- | --- | --- | --- | --- | --- | --- | --- | --- | --- | --- | --- | --- | --- | --- | --- | --- | --- | --- | --- |
|  | *InR* | *Pi3K* | *Akt* | *Punt* | *Babo* | *dSmad2* | *Torso* | *Ras* | *Raf* | *Erk* | *Nvd* | *Spo* | *Phm* | *Dib* | *Sad* | *Shd* | *EcR* | *Br-C* | *E74* |  |
| *C. suppressalis* | ☆ | - | - | ☆ | ☆ | ☆ | - | ☆ | ☆ | ☆ | ☆ | R | ☆ | ☆ | - | ☆ | R | - | - |  |
| *S. incertulas* | - | - | - | - | - | - | - | - | - | - | - | - | - | - | - | - | ☆ |  |  |  |
| *N. lugens* | ☆ | ☆ | R | ☆ | - | ☆ | ☆ | ☆ | ☆ | ☆ | - | - | ☆ | ☆ | ☆ | ☆ | M | ☆ | ☆ |  |
| *L. striatellus* | - | - | - | - | - | - | - | - | - | - | - |  | ☆ | R | ☆ | ☆ | ☆ | - | - |  |
| *S. furcifera* | - | - | - | - | - | - | - | - | - | - | - |  | R | R | ☆ | ☆ | ☆ | - | - |  |

Note: R, target gene predicted by RNAhybrid; M, target gene predicted by miRanda; RM, target gene predicted by both of RNAhybrid and miRanda; ☆, gene with 3’UTR but not predicted to be target of mir-14 by neither RNAhybrid nor miRanda; -, gene with no available 3’UTR sequence.

Table S4 Copy number and mRNA expression level of miR-14 detected in T_0_ generation transgenic rice plants.

| Plant ID | Copy number | Relative expression (mean ± sd)^£^ |
| --- | --- | --- |
| C#24 | 1 | 65.79 ± 1.47c |
| C#15 | 1 | 36.14 ± 12.44e |
| C#18 | 1 | 24.42 ± 5.05ef |
| C#27 | 1 | 18.17 ± 1.94fg |
| C#8 | 1 | 13.27 ± 0.68fg |
| C#30 | 1 | 11.53 ± 2.81fg |
| C#7 | 1 | 8.45 ± 5.09fg |
| C#16 | 1 | 7.34 ± 0.44fg |
| C#14 | 1 | 4.73 ± 0.41g |
| C#31 | 1 | 1.02 ± 0.24g |
| C#28 | 1 | 0.69 ± 0.08g |
| C#32 | 1 | 0.30 ± 0.08g |
| C#26 | 2 | 48.52 ± 4.32de |
| C#6 | 2 | 39.54 ± 1.34de |
| C#19 | 2 | 38.83 ± 6.92de |
| C#17 | 2 | 31.62 ± 5.36ef |
| C#1 | 2 | 24.53 ± 5.56ef |
| C#4 | 2 | 21.54 ± 2.00f |
| C#12 | 2 | 13.79 ± 3.00fg |
| C#29 | 2 | 9.70 ± 1.00fg |
| C#34 | 2 | 4.11 ± 0.91g |
| C#33 | 2 | 2.14 ± 0.36g |
| C#2 | 2 | 0.07 ± 0.01g |
| C#22 | 3 | 205.17 ± 9.40a |
| C#20 | 3 | 29.54 ± 4.82ef |
| C#11 | 3 | 6.58 ± 3.81g |
| C#35 | 3 | 1.91 ± 0.32g |
| C#25 | 4 | 149.02 ± 12.46b |
| C#13 | 4 | 26.58 ± 3.79ef |
| C#10 | 4 | 18.26 ± 0.85fg |
| C#3 | 4 | 16.43 ± 3.84fg |
| C#21 | 4 | 0.02 ± 0.01g |
| C#5 | 8 | 11.95 ± 3.17g |
| WT | 0 | 0.01 ± 0.00g |

£: Expression values were analyzed by One-way ANOVA with the multiple comparison of Tuekey’s test. Different letters indicate statistically significant difference (*p* < 0.05).

Table S5 Primers used in the study

| Purpose | Primer name | Primer sequence |
| --- | --- | --- |
| qRT-PCR | qCsSpo-F | TCCAACCTACAAACCAGACG |
|  | qCsSpo-R | ATCGCAGAGTTTGGATGAGG |
|  | qCsEcR-F | ATCGAAGACCTTCTGCACTTC |
|  | qCsEcR-F | CTGGCCGGTCTGAGAATATAAC |
|  | qCsAct-F | CAACGGATATCTCGGCTCT |
|  | qCsAct-R | CAACTTGCGTTCAAAGACTC |
|  | C-RT^£^ | GTCGTATCCAGTGCAGGGTCCGAGGTATTCGCACTGGATACGACATAGGA |
|  | N-RT^£^ | GTCGTATCCAGTGCAGGGTCCGAGGTATTCGCACTGGATACGACTAGGAG |
|  | URP^§^ | GTGCAGGGTCCGAGGT |
|  | miR-14-SP^§^ | cgcagcTCAGTCTTTTTCTCT |
|  | 18sRNA-F | TCGAGCCGCACGAGATTGAGCA |
|  | 18sRNA-R | CAAAGGGCAGGGACGTAATCAAC |
|  | OsU6-F | CAACGGATATCTCGGCTCT |
|  | OsU6-R | CAACTTGCGTTCAAAGACTC |
| Selective selection & southern blot | Hpt557-F | ACACTACATGGCGTGATTTCAT |
|  | Hpt557-R | TCCACTATCGGCGAGTACTTCT |

£: Universal primer for the reverse reaction in the stem loop qRT-PCR;

§: Universal reverse primers (URP) for the real-time PCR
